# Impact of Place Identity, Self-Efficacy and Anxiety State on the Relationship Between Coastal Flooding Risk Perception and the Willingness to Cope

**DOI:** 10.3389/fpsyg.2019.00499

**Published:** 2019-03-11

**Authors:** Colin Lemée, Ghozlane Fleury-Bahi, Oscar Navarro

**Affiliations:** Laboratoire de Psychologie des Pays de la Loire, University of Nantes, Nantes, France

**Keywords:** perceived risk, coastal flooding, coping willingness, anxiety-state, place identity, Bayesian model comparison

## Abstract

Inhabitants of coastal areas are constantly confronted with minor or major events such as storms, erosion or flooding. This article investigates the predictors of coping willingness among citizens exposed to coastal flooding. Coping can be defined as a set of cognitive and behavioral efforts to master, reduce or tolerate a given risk and these strategies are generally regrouped into two different categories: active coping strategies oriented toward the risk to reduce or master it, and passive coping strategies focused on the reduction of internal tensions such as anxiety or fear. In this paper, we focus especially on how place identity, perceived self-efficacy, anxiety-state and coastal flooding risk perception shape both active and passive coping willingness. Data were obtained from different areas at risk of coastal flooding located in France. The sample is composed of 315 adult participants (mean age = 47; *SD* = 15). Two competing models were tested using path modeling. We expected a direct relation between risk perception and the willingness to cope actively and that a higher perceived self-efficacy would increase active coping willingness. Concerning passive coping strategies, we expected that a higher anxiety-state increases passive coping willingness, and that place identity would act as a mediator and increases the relation between anxiety-state and passive coping willingness. Results suggest that place identity increased when the living place is threatened and that the use of passive coping strategies also increased. Also, we demonstrated a direct relation between risk perception and active coping willingness but it appeared that self-efficacy has no effect on this relation. Model fit indices suggest the good fit of our model and Bayesian model comparison reveals a very strong evidence of the best fit of this model compared to its saturated and independent equivalents.

## Introduction

### Coastal Flooding

In France only, approximately five million people are living in coastal areas concerned with coastal flooding (Kolen et al., 2010), i.e., a temporary flooding of the coastal area under severe weather and tide conditions (Chaumillon et al., 2017). Although rare, coastal flooding is a brutal phenomenon. Also, not only are coastal areas exposed to flood but there are also strong human and economic stakes (Meur-Férec et al., 2008). Especially, in coastal areas, the complexity of risk management is reinforced in cities that rely on tourism (Leichenko, 2011; Albers and Deppisch, 2013; Jabareen, 2013; Torabi et al., 2017).

Literature about natural risk management points the necessity of a fundamental transformation of policies and urban planning in order to accommodate and adapt to the increasing severity and frequency of extreme natural hazards (Hurlimann and March, 2012; Storbjörk and Hjerpe, 2014). Indeed, climate is undeniably changing and urban planning and policies still play a central role in the fight against climate change (Eraydin and Taşan-Kok, 2013). Even though some authors demonstrated a relationship between the proximity to the coastline and climate change belief (Milfont et al., 2014), these policies and plans are often rejected (Goeldner-Gianella, 2007; Goeldner-Gianella et al., 2015) or criticized for their unfairness and perceived injustice (Jarvie and Friend, 2016). Indeed, coastal flooding is usually addressed by building or adapting coastal defense structures or by relocating populations (Macintosh, 2013) and it is necessary to develop a more adaptive and proactive management (Torabi et al., 2018).

The Protection Motivation Theory (PMT) developed by Rogers (1975), is based on the work of Lazarus (1966) and accounts for one’s assessment of a threat probability and damage potential, as well as one’s assessment of its own ability to cope with, or avoid being harmed by the threat, considering the cost (or the difficulty) of these coping strategies. Adaptive and maladaptive coping strategies will result of these two different processes.

Although it was successfully applied by several authors to flooding (Grothmann and Reusswig, 2006; Bubeck et al., 2013; Poussin et al., 2014), the PMT does not account for the link among features of people’s relations with their living places and their “biased” environmental risk assessment had been empirically documented and theoretically framed within social-environmental psychology, at least for over 20 years, i.e., since Bonaiuto et al. (1996). It is clearly established in social and environmental psychology that the risk perception of non-experts will differ from experts’ risk perception. For example, Bonnes et al. (2007) noted that environmental policies, which are based on experts’ criteria, could be misunderstood and opposed by inhabitants of risk areas because they do not share the same criteria in their environmental assessment. In this perspective, it is necessary to take into account the psychological mechanisms underlying the shift from risk perception to the implementation of coping strategies among non-experts. It is a necessary step to overcome citizen rejection of actual risk management policies and to promote adapted risk management plans (Goeldner-Gianella, 2007; Goeldner-Gianella et al., 2015; González-Riancho et al., 2015).

For this reason, we chose to develop a model that focuses on risk perception and coping willingness that would take into account specific aspects of coastal areas, such as the strong identification of people to their living place (i.e., place identity) (Michel-Guillou and Meur-Ferec, 2016, 2017). The development of such model should also enlighten the complex relations between risk perception, place identity and coping willingness (De Dominicis et al., 2015; Bonaiuto et al., 2016).

### Psychometric Paradigm of Risk

The psychometric paradigm of risk was developed during the 1970s and 1980s by Slovic and his team. The main objective of this approach was to account for the perception of non-experts on environmental and technological risks that are complex, imperceptible and unpredictable (Fischhoff et al., 1978). In traditional models, risk is considered as a complex process whose aim is “to maximize earnings and minimize losses in order to account for the dangerousness of investments” (von Neumann and Morgenstern, 2007).

The psychometric paradigm seeks “to take into account and quantify individuals’ subjective opinions about risks” (Slovic, 1987). These works led to the identification of three higher order factors: the fear inspired by the risk, knowledge of risk and perceived risk exposure. Most of the risk assessment variability would be explained by the extent to which the risks would be assessed on these three characteristics (Fear, Knowledge, and Risk Exposure) (Slovic, 1992) according to psychological, social, cultural and political determinants (Fischhoff et al., 1978; Slovic, 1987; Sjöberg et al., 2004).

Many research studies relied on the psychometric paradigm and “it has virtually always been possible to demonstrate that the factor structure is fairly invariant” (Sjöberg et al., 2004, 16). Also, in the field of environmental psychology, Terpstra et al. (2009) used a scale based on psychometric paradigm to examine how communication about flooding is accompanied by changes in the perception of risk and perceived control toward the risk of flooding. The authors observed that participation in a workshop is not sufficient to produce statistically significant changes in the participants’ risk perceptions, even though they demonstrated higher level of perceived control right after the workshop.

Also, Michel-Guillou and Meur-Ferec (2016) revealed that even if inhabitants of areas at risk of coastal flooding seem to be well aware of this risk, it is perceived as moderate by individuals on areas at risk and is associated with a low level of personal vulnerability. It is considered to be of little concern with regard to the advantages provided by their living place, as they feel “very privileged to live in these areas,” in particular, a better quality of life and a highly valued identity (Michel-Guillou and Meur-Ferec, 2016, 20). For Rey-Valette et al. (2012), two different functions of beaches and shorelines coexists: a recreational function and a function of protection but it is as if the first one was fully assimilated by people while the second absolutely not. The relevance of psychometric approach on coastal flooding has already been demonstrated (Lemée et al., 2018). Indeed, the reassessment and adaptation of a scale developed for the assessment of flooding perception by Terpstra et al. (2006) revealed that a similar structure is found for coastal flooding risk (Lemée et al., 2018): Risk Exposure, Fear and Knowledge being the most important factors found in these studies in the perception of flooding and coastal flooding. Though, a more complex model that would account for the relations between coastal flooding risk perception and coping willingness remained necessary.

In particular, the possible impact of place identity on the relation between risk perception and coping willingness remained to be discussed, due to the lack of models in the literature that account for its impact in this particular context, where identity is clearly seen as valued by the living place.

### Understanding the Role of Place Identity

Place identity refers to the complex process of appropriating the living place as a personal identity. This appropriation is based on symbolic, emotional and social links between an individual and a place (Giuliani and Feldman, 1993; Williams and Vaske, 2003) and can be considered as a substructure of the self-identity (Proshansky et al., 1983): through personal experiences and attachment to a place, a person acquires a sense of belonging and purpose that give meaning to his life. In this sense, place identity “assumes that each city holds its own urban identity based on its main features” (Belanche et al., 2017). Thus, the city would become “a symbol of individual’s wealth in terms of personal experience that fits the classical functions (distinctiveness, stability, social value, etc.) attributed to identity” (Belanche et al., 2017). In the case of coastal areas, Lemée (2017) found that individuals are bonded emotionally to their living place and it is seen as valuing positively their identity. On the other hand, their place dependence [i.e., the capacity of a place to fulfill someone’s goals and needs better than another place (Lewicka, 2011)] seems relatively minor (Michel-Guillou and Meur-Ferec, 2016). Indeed, if coastal areas are seen as privileged, they do not offer more services or job opportunities than other places, especially in the case of minor coastal cities.

Moreover, as stated by Breakwell (2015), it is well-known that people cope with threats to their identities in many different ways. These coping strategies can generally be discussed at 3 different levels (intrapsychic, interpersonal, and intergroup). Also, in the case of coastal risks, these risks are a direct threat to both the living place and the place identity. Recent works by Meloni et al. (2019) showed the relevance of city identity and stress to explain the relationships between risk perception and the choice of coping strategies. It makes necessary to study how people will cope with this threat for their identity.

### Transactional Model of Stress and Coping

The concept of coping was proposed by Lazarus (1966) to account for “all the cognitive and behavioral efforts” that an individual will make to deal with a threatening event, in order “to master, control or tolerate its impact” on its “physical and/or psychological health” (Lazarus and Folkman, 1984, 843). These strategies are usually regrouped into two different categories: active coping strategies that would account for actions and behaviors oriented toward the problem in order to reduce or master the problem and passive strategies that would focus on negative emotions such as anxiety or fear in order to reduce internal tensions caused by the threatening situation. A lot of studies have demonstrated the impact of a natural hazard near the living place on physical and psychological health. The inability of individuals to fight effectively against environmental risks can lead to the development of a strong feeling of anxiety. This anxiety refers to a psychological and physiological state characterized by the anticipation of negative events. Thus, it differs from fear, as it is considered by the psychometric paradigm, which corresponds to the assessment of the severity of a risk, should this risk occurs. Then, this feeling of anxiety could be accompanied by the implementation of passive coping strategies focused on the reduction of these internal tensions (Lopez-Vazquez and Marvan, 2004). Beyond an immediate response of fear during a risky event, anxiety and anxious anticipation of such an event are likely to have an impact in the short and long term on the individual.

If we consider the transactional model of stress and coping developed by Lazarus (1966), the individual’s own assessment of their coping ability as inadequate, insufficient or unavailable would be responsible for such a state. Also, Panno et al. (2015) observed in their studies that the use of cognitive reappraisal (an active coping strategy) was linked to pro-environmental behavior and climate change perception, while cognitive suppression (a passive coping strategy) was not. Thus, we hypothesized that a high level of anxiety toward the risk would be accompanied by the implementation of passive coping strategies (i.e., strategies focused on the reduction of negative feelings and emotions) rather than active coping strategies. Moreover, we hypothesize that among individuals who exhibit greater anxiety about coastal flooding risk – which is a threat to their place of residence and their place identity – their place identity should increase, and it should also lead to a more important use of passive coping strategies, focused on emotions.

On the other hand, self-efficacy, as theorized by Bandura (1994) as the beliefs of individuals in their abilities to perform tasks or performances clearly echoes the secondary evaluation, theorized by Lazarus and Folkman (1984) as the individual’s estimate of its personal resources, in order to deal with a threat. We consider the possible impact of self-efficacy as a moderator between risk perception and active coping willingness. A high level of self-efficacy should then facilitate the transition from cognition to action: from risk perception to active coping willingness. Indeed, it refers to the belief of an individual that he would be able to engage in specific actions and contribute to a personal or collective goal with success.

In conclusion, we hypothesize that a high level of perceived risk should favor the implementation of active coping strategies, while a high level of anxiety-state should, on the contrary, promote the implementation of passive coping strategies. Moreover, place identity should act as a moderator between anxiety-state and passive coping and reinforce the willingness to use such strategies when the living place is seen as threatened by the risk.

### Aims and Expectations

**FIGURE 1:**
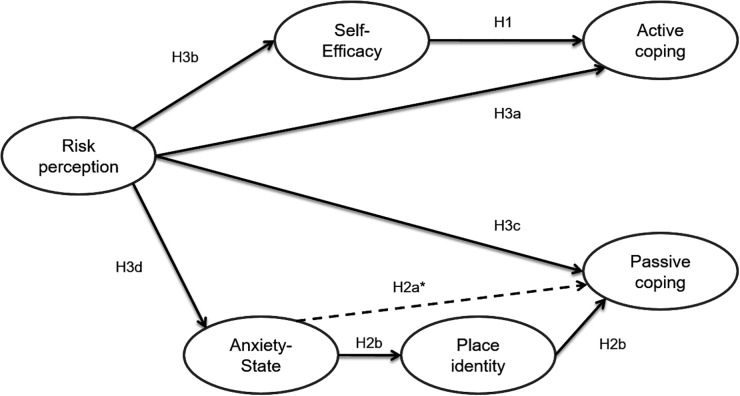
Path model of predicted causal effects. ^∗^The second model does not integrate Place identity. It considers only the direct relation between anxiety-state and passive coping willingness (H2a).

This article investigates the predictors of coping willingness among citizens exposed to coastal flooding. We focus especially on how place identity, perceived self-efficacy, anxiety-state and cognitive evaluation of coastal flooding risk are connected to both active and passive coping willingness. Figure 1 presents the expected relations among these variables.

To our knowledge there is no model that accounts for the relations between risk perception, coping willingness and place identity.

Beginning with active coping, the model reflects our expectation that a higher perceived self-efficacy will be correlated to active coping willingness (H1). Indeed, a high perceived self-efficacy should facilitate the transition from cognition to action and be correlated to the willingness to use active coping. Concerning passive coping strategies, we predict that a higher anxiety state will be correlated to passive coping willingness (H2a), and that this relation is mediated by the extent to which a person is identified to his living place (H2b). Indeed, we expect place identity to be correlated to the willingness to use passive coping strategies such as resignation, denial or avoidance, and to be predicted by individual anxiety toward the risk. To test this hypothesis, two different models will be tested and compared in order to determine the pertinence of place identity as a moderator between anxiety state toward the risk and passive coping willingness: a first model which integrates place identity and a second model that doesn’t integrate place identity.

Finally, for both active and passive coping, we expect direct and indirect effects of risk perception on active and passive coping willingness, as shown in Figure 1 (H3a; H3b; H3c; and H3d). Due to the high number of items in the different scales, the model displayed in Figure 1 will be tested using path modeling.

## Materials and Methods

### Sample and Procedure

Data were obtained from different areas at risk of coastal flooding located in France. The sample is composed of 315 adult participants (mean age = 47; *SD* = 15). The large proportion of seniors in our sample is representative of the over-representation of elderly people in the coastal areas in France (Vinet et al., 2012). Details related to the composition of the sample are presented in Table 1. Participants were asked for their postal code and whether they lived in the area at risk. All participants were living in areas at risk of coastal flooding, located in the western coastal region of France. Site selection was based on coastal risk prevention plans published by the French state. All sites were at equal risk. These plans represent areas exposed to a coastal flood phenomenon. On this basis, three areas at risk were finally selected because of their vulnerability to coastal flooding: Noirmoutier, Bay of Bourgneuf, and Guérande Peninsula (Préfecture de la Vendée, 2015; Préfecture de Loire Atlantique, 2016, 2017). These areas are all geographically close. They have in common to be oriented toward the sea and the summer tourism and they are towns of modest size (fewer than 15 000 inhabitants in 2014).

**Table 1:** Demographic Characteristics of the sample (*n* = 315).

	*n*	*%*	Mean	*s.d.*	
***Gender***					
Men	153	*48.5*			
Women	162	51.4			
Age			47 years	*15*	
*Location (in Pays de la Loire Region)*					
Noirmoutier	143	*45.4*			
Bay of Bourgneuf	105	*33.3*			
Guérande Peninsula	67	*21.3*			
**Duration of residency in the same city**			17.9 years	*15.4*	
*Professional status*					
Active	180	*57.1*			
Unemployed	18	*5.7*			
Retired	80	*25.4*			
Student	15	*4.8*			
At home	12	*3.8*			
Other situation	9	*2.8*			


*Italicized value means frequency and standard deviation.*

The survey was carried out online during winter and spring 2015. The duration for the questionnaire was 25 min for the whole questionnaire. All participants were volunteers and their responses were kept confidential and anonymous. In order to ensure the widest possible dissemination of the questionnaire, associations, local press, and public institutions were asked to relay the questionnaire link to their collaborators, members, etc., in the different areas concerned by the study.

Participants were instructed on how to complete the questionnaire before the statements. They were asked to respond to each question by selecting the answer that best reflected their opinion on a five-point scale, ranging from “completely disagree” to “completely agree.”

An ethics approval was not required for this study as per institutional and national guidelines and regulations. However, the study was carried out in accordance with University of Nantes ethics guidelines and the French law n° 2004-801 of August 6, 2004 relating to the protection of the natural persons with regard to the processing of personal data and amending Law No. 78-17 of 6 January 1978 relating to data, files and freedoms. Ethics was checked at the laboratory level. Participation in the research was voluntary, and the data were collected in an anonymous form. Online informed consent was obtained from all participants. Participants had to check a box on the online form in order to consent to the research.

### Material

The questionnaire was composed of five different scales: risk perception, coping, place identity, anxiety-state and self-efficacy. The coping and place identity scales were translated by a bilingual person and two researchers in the field of environmental social psychology. For every item, the different versions were discussed in order to retain the best translation.

In order to assess the coastal flooding risk perception, we chose to rely on the CFRES scale developed for the assessment of this specific risk (Lemée et al., 2018). Based on the works of Terpstra et al. (2006), this scale was validated on two different samples of inhabitants of areas at coastal flooding (the first one, also used in this study, in Metropolitan France and the second one in Guadeloupe, a French Caribbean island). It takes into account four different dimensions in the assessment of the risk of flooding by the sea: “*Risk Augmentation*” (F1) (“Due to climate change, the risk of flooding by the sea will increase considerably”), “*Perceived Vulnerability for Self*” (F2) (“Living near the ocean is a threat to my safety”), “*Collective Vulnerability*” (F3) (“It is necessary to strengthen the coast protections”) and “*Unknown Risk*” (F4) (“I can estimate the probability of a coastal flooding risk”). The whole process of development, adaptation and validation is presented in Lemée et al. (2018). In this study, a CFA revealed the good fit of the measurement model (RMSEA = 0.062; CFI = 0.937; TLI = 0.922; GFI = 0.928; SRMR = 0.069). F1 had an alpha of 0.81, F2 presented an alpha of 0.86, F3 presented an alpha of 0.71 and F4 an alpha of 0.64. A threshold of 0.7 is often seen as necessary, though Cronbach’s alpha is a coefficient of consistency and a value superior to 0.6 can be considered as satisfactory (Schmitt, 1996; Loewenthal and Lewis, 2015). This is why, for theoretical reasons we chose to keep this fourth factor in our analysis.

In order to assess the willingness to cope with coastal flooding risk, we chose to rely on the coping scale developed by Lopez-Vazquez and Marvan (2004). This scale has indeed been used in various environmental risk studies, for both natural and technological risks and showed a good reliability (Lopez-Vazquez and Marvan, 2004, 2012; Ruiz and Hernandez, 2014; Navarro et al., 2016). It consists of 26 items related to active and passive coping strategies. The scale adapted well to our context with a good internal consistency of its passive coping dimension (α = 0.710) and active coping (α = 0.872). Active coping was evaluated by items such as: “I’m gathering information from people who know the problem,” “I make changes in my environment to avoid a disaster,” “I ask professionals about the problem.” On the other side, passive coping was evaluated by items such as: “I refuse the idea that this situation is serious,” “I am carrying out activities to think of something else,” “I try not to think about the problem.”

We chose to use the sub-dimension “place identity” of the scale developed by Williams and Vaske (2003). The sub-dimension is composed of six items and the factorial analysis revealed a good internal consistency (α = 0.958). Among the different items of this scale, participants were asked: “I feel that my city is like a part of myself,” “My city is a very special place for me,” “I am very attached to my city.”

The State-Trait Anxiety Inventory (STAI-Y) developed by Spielberger et al. (1983) was chosen to report participants’ anxiety toward coastal flooding risk. The French version of the STAI-Y was used, in its translation by Spielberger et al. (1993). Among the different items of this scale, participants were asked how they felt in the case of a possible coastal flooding : “I feel calm,” “I am confident,” “I am worried about possible misfortunes.” Even though the STAI-Y gives a score for each participant and is not scaled on a 5-point Likert scale, which limits its direct interpretation in regard to the other indicators, it is an extremely reliable scale and it has been used in a very large number of publications, i.e., more than 2000, according to Spielberger et al. (1993). It consists of 20 items. These considerations led us to retain this scale.

In order to account for the participants’ self-efficacy, we developed a three-item scale, inspired by previous works of Demarque et al. (2011); Lheureux et al. (2011), and Navarro et al. (2016) in the field of environmental risks perception. These items were: “My choices to deal with coastal flooding are numerous” “With my knowledge of coastal flooding, I am really able to act” and “If I were more involved in coastal flooding problems, that would change a lot of things.” The factor analysis reveals a good internal consistency of this tool (α = 0.82).

To answer the different scales participants had to indicate on a 5-point Likert scale their degree of agreement to each item. Mean value was used for the different scale, except for the anxiety-state scale for which a global score was computed. This score goes from 20 to a maximum of 80 and, according to Spielberger et al. (1993) a score between 46 and 55 corresponds to “average anxiety.”

### Data Analysis

Because they were no model that clearly established how risk perception, place identity and coping willingness are tied together, we chose to develop and to compare two different models that would enlighten these relations in the context of coastal flooding risk. The first model integrates place identity as a moderator between anxiety and passive coping while the second does not integrate place identity. Descriptive analysis and analysis of variance were performed using SPSS 22 (IBM Corporation, 2013b). Path analysis was performed using AMOS 22 (IBM Corporation, 2013a). There were no missing data. Descriptive statistics are provided in Table 2 and the correlation matrix between the different variables in Table 3. Before the analysis, the data were checked for normality. Two steps lead the analysis. First, the direct and indirect relations between risk perception and coping willingness were examined using path models and standardized path coefficients for the two different models (Schumacker and Lomax, 2010). This technique is well adapted for small samples (Shrout and Bolger, 2002). A maximum likelihood method was used. Different fit indices were examined to evaluate the path model’s fit: the chi-square, the goodness-of-fit index (GFI), the comparative fit index (CFI), the Tucker-Lewis index (TLI), and the root mean square error of approximation (RMSEA). It is accepted the chi-square must not be significant but it is very dependent of the size of the sample (Pui-Wa and Qiong, 2007). This is why Wheaton et al. (1977) suggest that the researcher also computes a relative chi-square (χ^2^/df or CMIN/df). A χ^2^/df ratio < 3.00 is satisfactory. The GFI, CFI, and TLI must be greater than 0.90 and the RMSEA must be less than 0.05 (Schumacker and Lomax, 2010). As a second step, we used Bayesian criteria to decide if the model needed adjustment and we compared the two different models M^1^ and M^2^ to their independent and saturated equivalent and compared M^1^ to M^2^ (Raftery, 1995; Lee and Wagenmakers, 2014). The Bayesian Information Criterion (BIC) delivers information on the quality of adjustment of a model to the data, which is measured by the likelihood. Also, it takes into account the complexity of the model (measured by its number of unknown parameters) and retains the model that achieves the best compromise between quality of fit and parsimony. The absolute value of the BIC must be as close as possible to 0 (Noël, 2013) and according to Raftery (1995) it is possible to determine the extent to which a model is better than another model using the BIC absolute value. In our case, we expected M^1^ that integrates place identity to be more probable than M^2^.

**Table 2:** Means, standard deviation, skewness and kurtosis for each variables.

	n	mean	*SD*	Skew.	Kurt.	Min.	Max.
Place identity	315	3.5	1.28	0.58	0.57	1	5
Self-efficacy	315	2.53	*0.8*	0.28	0.03	1	5
Risk perception	315	3.27	*0.3*	0.33	0.05	2	4
Anxiety-State	315	53.11	13.35	1.4	2.78	20	76.00
Active coping	315	3.36	*1.3*	0.7	0.4	1	5
Passive coping	315	2.6	*0.5*	0.28	0.09	1	4


*Italicized value means standard deviation.*

**Table 3:** Means and standard deviation for each variables on the different sites investigated.

		Noirmoutier	Bay of Bourgneuf	Guérande Peninsula
Place identity	*M*	3.51	3.60	3.47
	*SD*	*1.14*	*1.12*	*1.29*
Anxiety-State	M	51.72	54.08	54.58
	*SD*	*14.32*	*12.76*	*11.93*
Risk Perception	M	3.30	3.25	3.22
	*SD*	*0.36*	*0.36*	*0.35*
Active Coping	M	3.25	3.32	3.42
	*SD*	*0.91*	*0.89*	*0.78*
Passive Coping	*M*	2.53	2.69	2.61
	*SD*	*0.55*	*0.54*	*0.57*
Self-efficacy	*M*	2.61	2.52	2.39
	*SD*	*0.87*	*0.85*	*0.85*


*Italicized value means standard deviation.*

## Results

### Descriptive Statistics

For the whole sample (*n* = 315), the average risk assessment score was 3.27 (*SD* = 0.3). It can be considered as a moderate risk assessment. Similarly, the average anxiety score was 53.11 (*SD* = 13.35). According to the thresholds defined by Spielberger et al. (1993), a score between 46 and 55 corresponds to “average anxiety.”

Concerning place identity, place identity scores are rather high (*m* = 3.5, *SD* = 1.28).

Concerning coping willingness, the participants declare more willingness for active coping strategies (*m* = 3.36, *SD* = 1.3) than for passive coping strategies (*m* = 2.06, *SD* = 0.5) [*t*(314) = 83.87, *p* < 0.001]. Details are presented in Table 2.

Also, descriptive results for the main variables revealed no differences between the three different sites (Table 3). A correlation matrix is presented in Table 4 for the main variables. It supports our expectations that risk perception is correlated to active coping and self-efficacy, and that passive coping is linked to both place identity and anxiety.

**Table 4:** Correlation matrix of the relations between place identity, self-efficacy, risk perception, anxiety-state and active and passive coping.

	Place identity	Self-efficacy	Risk perception	Anxiety-State	Active Coping	Passive coping
Place identity	—	*0.11*	*0.04*	*0.26*^∗∗∗^	*0.14*	*0.23*^∗∗∗^
Self-efficacy		—	0.40^∗∗∗^	0.16	0.07	0.08
Risk perception			—	0.31^∗∗∗^	0.30^∗∗^	0.02
Anxiety-State				—	0.04	0.22^∗∗∗^
Active coping					—	–0.11
Passive coping						—


*^∗∗^p < 0.005, ^∗∗∗^*p* < 0.001.*

### Model Fit and Model Comparison

Model fit indices suggest a good model fit of M^1^ which integrates place identity (RMSEA = 0.054; CFI = 0.95; IFI = 0.96; NFI = 0.92), but, because of a significant χ^2^ [χ^2^ (8) = 23, 42, *p* < 0.001], we examined the CMIN/df which can be considered as correct (CMIN/df = 1.9).

**FIGURE 2:**
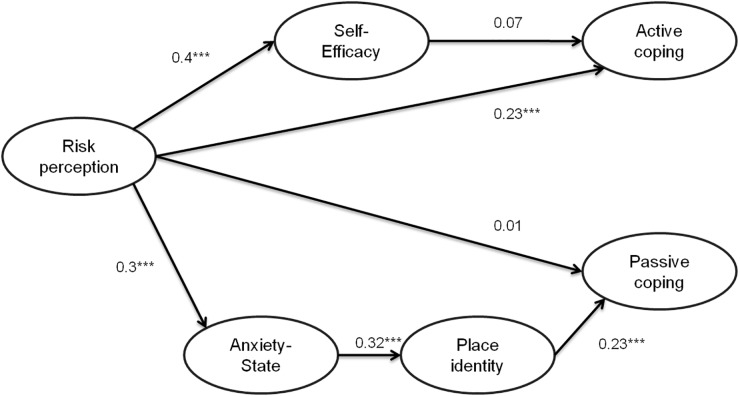
Path modeling results for M^1^. (1) Reported numbers are standardized regression coefficients (β) indicating direct effects. (2) *N* = 315 4. ^∗∗∗^*p* < 0.001.

The analysis did not support the predicted effects of self-efficacy on active coping (H1; β = 0.07, *p* = 0.24). But it supports the effect of anxiety-state (H2a; β = 0.32, *p* < 0.001) on the place identity and place identity on passive coping (H2a; β = 0.23, *p* < 0.001). Finally, it supports the effect of risk perception on self-efficacy (H3a; β = 0.4, *p* < 0.0001) and on active coping (H3b; β = 0.23, *p* < 0.001) and the effect of risk perception on anxiety-state (H3d; β = 0.3, *p* < 0.001). But the effect of risk perception on passive coping was not confirmed (H3c; β = 0.01, *p* = 0.99). The results of this procedure are depicted in Figure 2.

The results showed that M^1^ (BIC M^1^ = 54.4) has a BIC value lower than the saturated model (BIC M^1SAT^ = 65.3) and the independent model (BIC M^1IND^ = 175.9), the difference between M^1^ and M^1SAT^ (M^1SAT^ – M^1^ = 10.9) and between M^1^ and M^1IND^ (M^1IND^ – M^1^ = 120.9) is superior to 10. According to Raftery (1995) it means a very strong evidence of the better fit of the model M^1^. According to Lee and Wagenmakers (2014) this difference means a moderate evidence of the better fit of the model M^1^.

Model fit indices suggest a poor model fit of M^2^ (RMSEA = 0.079; CFI = 0.91; IFI = 0.92; NFI = 0.88). Indeed, RMSEA is higher than 0.05 and NFI is lower to 0.90 which is the lowest threshold. Because of a significant χ^2^ [χ^2^ (8) = 20, 8, *p* < 0.005], we examined the CMIN/df. With a value of 3, it can be considered as correct. Also, the results showed that M^2^ (BIC M^2^ = 61.7) has a BIC value higher than the saturated model (BIC M^2SAT^ = 55.2) and lower than its independent equivalent (BIC M^2IND^ = 192.4). Due to the poor adjustment of M^2^ and the poorer fit of the M^2^ model compared to its saturated equivalent (M^2^ – M^2SAT^ = 6.5), it appears that this model does not correctly describe the data.

The comparison between the models M^1^ and M^2^ (M^1^ – M^2^ = -7.3) also reveals that M1 is the most probable model.

## Discussion

Coastal areas are particularly vulnerable to climate change and hazards (Adger et al., 2005) and it is exacerbated in cities that rely on tourism (Albers and Deppisch, 2013). The need of a better understanding of people’s rejection of actual coastal risk management policies and the necessity to enlighten the relations between risk perception, coping willingness and place identity led us to propose a model that would take into account specific aspects of coastal areas.

Descriptive results confirm our expectations. Indeed, we observe a moderate risk perception and a moderate anxiety-state toward coastal flooding. It is consistent with early works on this topic and general literature about spatial bias in environmental risk assessment. Indeed, inhabitants of coastal areas at risk do not feel particularly worried about coastal risk (Rey-Valette et al., 2012; Michel-Guillou and Meur-Ferec, 2016) and different bias could explain these results, especially at a proximal level (Milfont et al., 2011). Concerning place identity, according to our expectations, the participants are identified to their living place. Considering the lack of consensus in the literature about the links between risk perception and place identity, this result seems particularly interesting in regards to coastal risk management and can potentially be extended to other risk areas that offer a valorization of identity. We also observe a higher level of willingness for active coping strategies, compared to passive coping willingness. Such a result may seem counterintuitive. When facing environmental threats which usually leave them helpless, individuals tend to favor passive coping strategies (Lopez-Vazquez and Marvan, 2004), but in our case, participants reported low levels of anxiety-state and risk perception toward the risk. The need for passive strategies is therefore reduced. Also, authors have observed that threatening information about climate change may have positive effects on pro-environmental behaviors to a certain point (Uhl et al., 2016). It is as if beyond a certain threshold, information perceived as too threatening would have undesired consequences on the willingness to carry out and maintain certain behaviors and would favor passive coping. In our case, the low level of risk perception may be sufficient to encourage the use of active strategies without such side effects.

Finally, the fit of the proposed model M^1^ sheds some light about the complex relations between these variables. To discuss the implications of this model, two different aspects should be examined. Firstly, it seems to indicate that the relations between risk perception, active coping willingness and self-efficacy are not clear, in the sense that risk perception is linked to both self-efficacy and active coping but that self-efficacy and active coping are not linked. On the other hand, this model confirms our hypothesis concerning passive coping willingness. Indeed, the implementation of passive coping strategies seems to be linked to the level of anxiety-state toward the risk, and that place identity act as a moderator between these two variables.

Concerning self-efficacy, its role is not clearly established. Even if the relation between risk perception and self-efficacy is significant, there is no relation between self-efficacy and active coping willingness. We can make different assumptions to explain this result. It may be interesting to introduce a wider range of self-efficacy measures into this model. Indeed, we may argue that coastal flooding is a collective problem that should be addressed through different efficacy belief levels (Lubell, 2002; Bonniface and Henley, 2008). As observed by Homburg and Stolberg (2006) and Chen (2015), collective efficacy is a better predictor of pro-environmental behaviors than self-efficacy and can be linked to an active problem coping to answer environmental challenges. Indeed, in their studies, Homburg and Stolberg (2006) also found that self-efficacy did not predict problem-focused coping. To improve our model, it would be necessary, then, to include self-efficacy beliefs and, on a higher level, collective efficacy beliefs, and institutional efficacy beliefs. Another way to address this finding would be to consider specific protective behaviors in addition to coping willingness.

About our second path, the implementation of passive coping strategies seems to be linked to the level of anxiety-state toward the risk, and that place identity acts as a moderator between these two variables. In other words, it suggests that place identity is higher when the living place is perceived as threatened and it correlates with the willingness to use passive coping strategies. Such considerations, if proven true by further studies should be taken into account by coastal risk managers and risk management policies.

## Conclusion

In conclusion, as we said before, the good fit of the proposed model is encouraging and, concerning coastal management, it delivers crucial hints for coastal risk management. Indeed, if proactive and participative management are recommended, our model suggests that a stronger anxiety-state toward the risk is accompanied by a higher level of identification to the living place, which is correlated to the implementation of passive coping strategies. To our knowledge, this is an unprecedented model. It suggests the relevance of place identity within a study on coastal risk perception. On the other hand, the transition from cognition to action is not clear among the inhabitants with a high level of perceived self-efficacy. Their self-efficacy beliefs are not systematically accompanied by higher level of active coping willingness.

Different improvements can be considered in order to pursue this work. The relations between place identity, risk perception and coping willingness are known to vary depending on the place specificities (Bernardo, 2013; Casakin et al., 2015). According to Vinet et al. (2012), two different types of coastal cities should be distinguished: we focused here on coastal cities whose activity is mainly linked to tourism and it is possible these relations would differ in coastal cities where traditional activities persist. Moreover, based on our findings, the role of place identity as a mediating variable should be investigated for other risk studies in locations where identity is perceived as valued by the place.

Also Raymond et al. (2017) suggests that “place attachments and place meanings are slow to evolve” which in turn prevent behaviors to evolve (including protective behaviors). In that sense, the affordance theory could “complement (these) slower forms of social construction” and could play a role for supporting behavior changes (Raymond et al., 2017; Carrus et al., 2018). Thus, to develop an approach based on a direct perception of possible behaviors or opportunities in the environment should be a good way to support change and to promote adapted protective behaviors in coastal areas.

Another way to improve our model would be to introduce a list of actual defense behaviors against coastal flooding risk, instead of a coping willingness scale. On the one hand, it is possible that it enlightens the relations between risk perception, self-efficacy and defense behaviors. On the other hand, it would provide a more precise understanding of the defense strategies used against coastal flooding risk.

Finally, it is possible that social desirability explains the difference between active and passive coping willingness. Especially when we consider the important legislative activity under way in these municipalities and the implementation of public risk management policies, declaring passive coping willingness might appear as a less suitable response for participants. In this study, though, such desirability bias does not have a significant impact on the development and validation of a risk perception and coping model, which seeks only to determine the nature of the relations between these variables, but future studies should take into consideration this particular kind of bias. Also, this study relied on a correlational design. Further studies should replicate these findings to ensure their validity. In particular, similar results concerning the relationships between anxiety, place identity and passive coping would be extremely important to ensure the relevance of the findings presented here, in the specific context of coastal areas.

## Author Contributions

CL contributed in data collection and analysis, plus conception of study and writing of the manuscript. ON and GF-B contributed to the conception of the study and review of the manuscript and analysis.

## Conflict of Interest Statement

The authors declare that the research was conducted in the absence of any commercial or financial relationships that could be construed as a potential conflict of interest.
